# Identification of serum biomarkers for aging and anabolic response

**DOI:** 10.1186/1742-4933-8-5

**Published:** 2011-06-20

**Authors:** Camellia Banerjee, Jagadish Ulloor, Edgar L Dillon, Qusai Dahodwala, Brittani Franklin, Thomas Storer, Paola Sebastiani, Melinda Sheffield-Moore, Randall J Urban, Shalender Bhasin, Monty Montano

**Affiliations:** 1 Section of Infectious Diseases, Department of Medicine, Boston University School of Medicine, 710 Albany Street, Boston MA, 02118, USA; 2 Section of Endocrinology, Diabetes and Nutrition, Department of Medicine, Boston University School of Medicine, 710 Albany Street, Boston MA, 02118, USA; 3 Department of Biostatistics, Boston University School of Public Health, 715 Albany Street, Boston, 02118, USA; 4 Division of Endocrinology and Metabolism, Department of Medicine, University of Texas Medical Branch, 301 University Boulevard, Galveston TX, 77555, USA

**Keywords:** Testosterone, Age, Biomarker

## Abstract

**Objective:**

With the progressive aging of the human population, there is an inexorable decline in muscle mass, strength and function. Anabolic supplementation with testosterone has been shown to effectively restore muscle mass in both young and elderly men. In this study, we were interested in identifying serum factors that change with age in two distinct age groups of healthy men, and whether these factors were affected by testosterone supplementation.

**Methods:**

We measured the protein levels of a number of serum biomarkers using a combination of banked serum samples from older men (60 to 75 years) and younger men (ages 18 to 35), as well as new serum specimens obtained through collaboration. We compared baseline levels of all biomarkers between young and older men. In addition, we evaluated potential changes in these biomarker levels in association with testosterone dose (low dose defined as 125 mg per week or below compared to high dose defined as 300 mg per week or above) in our banked specimens.

**Results:**

We identified nine serum biomarkers that differed between the young and older subjects. These age-associated biomarkers included: insulin-like growth factor (IGF1), N-terminal propeptide of type III collagen (PIIINP), monokine induced by gamma interferon (MIG), epithelial-derived neutrophil-activating peptide 78 (ENA78), interleukin 7 (IL-7), p40 subunit of interleukin 12 (IL-12p40), macrophage inflammatory protein 1β (MIP-1β), platelet derived growth factor β (PDGFβ) and interferon-inducible protein 10 (IP-10). We further observed testosterone dose-associated changes in some but not all age related markers: IGF1, PIIINP, leptin, MIG and ENA78. Gains in lean mass were confirmed by dual energy X-ray absorptiometry (DEXA).

**Conclusions:**

Results from this study suggest that there are potential phenotypic biomarkers in serum that can be associated with healthy aging and that some but not all of these biomarkers reflect gains in muscle mass upon testosterone administration.

## Introduction

As the general population ages, there is an increased prevalence of loss in muscle mass, raising the risk for frailty, declines in functional mobility, and early mortality [1-4]. Loss of lean muscle can also be a comorbid condition in multiple chronic and acute disorders including cancer cachexia, HIV-associated weight loss, inflammatory sepsis, and age-associated sarcopenia [5-8]. Biomarkers for healthy aging identifiable in the serum would be of substantial use in detecting age associated morbidities and initiating therapeutic pro-anabolic treatment.

Anabolic supplementation is broadly recognized to increase muscle mass in both elderly and young individuals [9,10]. Testosterone displays a dose dependent effect on gains of lean muscle mass and cross-sectional fiber area in both older and younger men [6,9,11-15]. However, because in some populations testosterone administration poses undesirable side effects, there is a motivation for identifying alternative, broadly effective anabolic therapeutics, such as selective androgen receptor modulators (SARMs) that improve muscle mass and physical function [16-18]. Therefore, the utility of serum biomarkers would help to gauge pro-anabolic activity of SARMs and therefore be of substantial value in clinical research to ameliorate declines in muscle mass and function.

In this study we were interested in identifying age-associated biomarkers for healthy aging and evaluating whether biomarkers that differ between healthy young and older men at baseline, also differ in response to graded doses of testosterone. To achieve this, we used banked serum specimens from younger and older men to measure selected soluble cytokines and growth factors, based on predicted biomarkers from previous studies by us and others [2,15,19-25]. Herein, we report results from this pilot analysis to identify biomarkers that change either in association with age and/or testosterone dosage.

## Methods

### Sample Population (Boston, MA)

The samples used for this study were obtained from a previously reported double-blind, randomized study that consisted of a 4-week control period, 20-week treatment period and 16-week recovery period [11,13]. Participants included sixty young men (age range 18 to 35 years) and sixty-one older men (age range 60 to 75 years). All subjects provided informed written consent according to protocol approved by the Charles Drew University and Research and Education Institute. Exclusion criteria included 1) presence of prostate disease defined as cancer, an American Urological Association symptom score of greater than 7, a prostate-specific antigen level greater than 4 ng/ml, 2) hematocrit above 48%, 3) diabetes mellitus, 4) heart problems including myocardial infarction or congestive heart failure all measured using a 12-lead electrocardiogram monitoring to exclude symptoms present during exercise as well, 5) severe sleep apena, 6) administration of androgenic steroids in the past year, 7) participation in sports events, resistance training or moderate to heavy endurance exercise training and 8) baseline testosterone levels below 300 ng/dL. For more in depth description of enrollment criteria and physical function, see Bhasin et al, in [11,13]. Stored serum samples at baseline and after treatment were used from 20 of the younger men and 19 of the older men based on availability. Mean baseline testosterone levels for younger men were 586 ng/dL and 358 ng/dL for older men.

### Sample Population (Houston, TX)

Stored baseline serum samples were used from 20 older men (age range 60 to 85 yrs) recruited through the Sealy Center of Aging Volunteer Registry at the University of Texas Medical Branch (UTMB) in Galveston, TX for inclusion in a randomized double-blinded placebo-controlled testosterone intervention study. All subjects provided informed written consent according to the guidelines established by the UTMB institutional review board and were medically screened. Qualified subjects had endogenous testosterone concentrations below 500 ng/dL and were otherwise healthy. To assess medical eligibility, subjects underwent a battery of tests including a history and physical examination, complete blood count, metabolic panel including fasting serum glucose and insulin, an electrocardiogram (ECG), plasma electrolytes, prostate specific antigen (PSA), liver and renal function and lipid panel. Subjects were included based upon their ability to provide regular transportation to the Clinical Research Center (CRC) at UTMB. Subject exclusion criteria included the following: 1) serum testosterone > 500 ng/dL, 2) indication of cardiovascular disease or heart problems assessed via a resting ECG and a Bruce protocol exercise stress test, 3) previous history of angina or myocardial infarction, 4) PSA > 4.0 μg/L, 5) history of prostate cancer, 6) history of severe benign prostatic hypertrophy, 7) LDL > 200 mg/dL, 8) hematocrit > 51%, 9) hypertension (>140/90 mmHg), 10) BMI > 35, 11) history of hepatitis or 3 × elevation of Alk phos, ALT, AST, 12) illnesses including diabetes, cancer, COPD, sleep apnea or any other causing disability, 13) bone related disorders, 14) DEXA lumbar score > -2.5, 15) currently taking Coumadin, glucocorticoids, androgens, or anti-bone-resorptive agents, and 16) regular physical exercise. These inclusion/exclusion criteria reflect those recommended by the Clinical Guidelines Subcommittee Task Force of The Endocrine Society[26] and previously published trials with testosterone and older men [27,28]. Mean baseline testosterone levels for these older men were 320 ng/dL.

### Testosterone supplementation (Boston, MA)

Serum samples were obtained from men who participated in a randomized testosterone supplementation trial. Men were treated with monthly injections of a long-acting GnRH agonist (Lupron depot, 7.5 mg; TAP, North Chicago, IL) to suppress endogenous testosterone production, and concomitantly weekly injections of one of five doses of testosterone enanthate (Delastryl, Savient Pharmaceuticals, NJ) [11]. Based on dichotomous functional outcomes in previous reports, testosterone doses were categorized as low (i.e., 25 mg, 50 mg, and 125 mg) and high (i.e., 300 mg and 600 mg).

### Biomarker measurements

The serum specimens were selected based on quality and availability. Quality was determined by visual inspection. Serum specimens for both populations had been collected, centrifuged and stored at similar conditions in both places. Serum factors were measured at two time intervals: early in the study, i.e., at baseline or within the first two weeks of starting GnRH and testosterone treatment, and later in the study, i.e., twenty weeks after initiation of GnRH and testosterone treatment. Insulin-like growth factor I (IGF1) was measured using an enzyme-linked immunosorbent assay (ELISA) using a non-extraction IGF-1 ELISA kit (Diagnostic Systems Laboratories, TX) in both young and old at baseline and after treatment. Pro-collagen III N-terminal peptide (PIIINP) was measured using validated equilibrium radioimmunoassay (RIA) (Orion Diagnostics, Espoo, Finland) as described previously[15,29] in both the young and the old subjects at baseline and after testosterone supplementation.

The remaining serum factors were measured using a multiplex Luminex platform (Panomics, Fremont, CA) as described previously [30]. This assay uses xMAP technology, a multi-analyte profiling Luminex technology, to detect and quantify multiple protein targets. The samples were run on a LiquiChip (Qiagen) and were analyzed using Qiagen Liquichip Analyzer software (Version 1.0.5.17455). A 35-plex was run on serum from the young men measuring ENA78, Eotaxin, FGF Basic, G-CSF, GM-CSF, GRO-α, IFNγ, IL1α, IL1β, IL-10, IL-12(p40), IL-12(p70), IL-13, IL-15, IL-17, IL-17F, IL-1RA, IL-2, IL-4, IL-5, IL-6, IL-7, IL-8, IP10, Leptin, MCP-3, MIG, MIP1α, MIP1β, NGF, PDGF-BB, RANTES, TNFα and TNFβ at baseline and after testosterone treatment. For those biomarkers that showed a change with testosterone treatment in the young, another 3-plex was run on serum from the older men treated with testosterone at baseline and after treatment measuring leptin, MIG and ENA78. A 30-plex assay was run to measure baseline cytokine levels of a separate group of older men before treatment measuring eotaxin, FGF basic, GCSF, GM-CSF, GROα, IFNγ, IL1α, IL1β, IL-10, IL-12(p40), IL-12(p70), IL-13, IL-17A, IL-2, IL-4, IL-5, IL-6, IL-7, IL-8. IP-10, MCP-1, MCP-3, MIP-1α, MIP-1β, NGF, PDGF-BB, RANTES, TNFα, TNFβ, and VEGF. Values outside of the range of the standard curve were omitted for the multiplex assays. The lower limit of detection for these analytes was 1 pg/ml. The CV range for inter-assay variability for the analytes was 6.73% - 17.25%, with an average of 12.24%.

### Statistical Methods

Baseline values of biomarkers were compared between younger and older men using a parametric two-sample t-test assuming unequal variances and a non-parametric two-sample Wilcoxon rank-sum test giving similar p-values. The determination of significance in biomarker response was based on a matched pair analysis of early versus late levels, and a bivariate categorical analysis of testosterone dose (low versus high) and age (younger men versus older men), using a parametric two-sample t-test with unequal variances and a non-parametric two-sample Wilcoxon rank-sum test with similar results. Statistical analyses were carried out in STATA version 8.0 (Stata Corp, College Station, Tex) and JMP 8.0.2 (SAS Institute Inc, Cary, NC). Values shown are all displayed as the mean plus/minus standard deviation unless otherwise indicated. Box plots are shown as quantiles, with the median (line in box), quartile range (edges of box), and extremes (vertical lines and points). In single-plex assays, siginificance was set at p < 0.05. In multiplex assays, a p-value < 0.05 was used to denote statististical significance while a p-value < 0.05/10 = 0.005 was used to take into account multiple comparisons using Bonferrorni correction.

## Results

### Study Population Characteristics

The study population consisted of healthy younger men (18-35 years old) and healthy older men (60-75 years old). We screened selected serum chemokines, cytokines, growth factors and angiogenic factors that we expected to change with age and possibly testosterone administration based on previous studies of aging and anabolic supplementation [2,15,19-24]. Serum samples were screened within the first two weeks of testosterone treatment and at week 20, the end of treatment period, to look for changes associated with testosterone dosage. Summary characteristics of the sampled population of younger and older men used for our serum data are shown in Table 1.

**Table 1:** Characteristics of study population

	Young	Old	P value
**Age (yr)**	27 ± 5 *(53)*	68 ± 7 *(39)*	<0.001
**Height (cm)**	175 ± 7 *(53)*	175 ± 7 *(39)*	0.9683
**Weight (kg)**	74.9 ± 10.0 *(53)*	84.3 ± 13.1 *(39)*	0.0003
**Body mass index (%)**	24 ± 3 *(53)*	27 ± 4 *(39)*	<0.001
**Serum total testosterone (ng/dl)**	586 ± 190 *(53)*	339 ± 95 *(39)*	<0.001
*Population Houston, TX*	N/A	358 ± 86 *(20)*	N/A
*Population Boston, MA*	586 ± 190 *(53)*	320 ± 103 *(19)*	N/A
**Lean Body Mass (mg/cm^2^)**	57336 ± 7209 *(52)*	58545 ± 7033 *(39)*	0.4245

Baseline characteristics of subjects evaluated in this study are shown. The number of subject used for each baseline value is indicated in parenthesis. The values are displayed as plus/minus standard deviation. The p-values are based on a Student *t*-test.

### Age related serum profile

**Figure 1:**
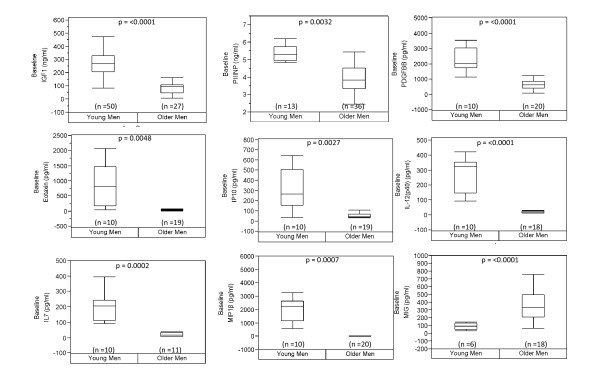
**Baseline levels of biomarkers in young and old men**. We evaluated a number of serum cytokines and chemokines in banked samples of older and younger men at baseline. Nine factors showed a significant change with age at these two groups (p-values from Student *t*-test < 0.05), and maintained statistically significant differences even after correcting for multiple comparison (p-value < 0.005).

The results of our multiplex assay identify nine serum proteins that differed (significance p < 0.005) between the two age groups, as shown in Figure 1. Eight of the nine serum proteins decreased with age: IGF-1, PIIINP, MIP-1β, IP-10, IL-7, IL-12p40, PDGFβ and eotaxin. The ninth serum protein, MIG showed higher levels in the older men relative to the younger men. All other analytes were below detection or not significant. These pilot data suggest that serum profiling may be a useful strategy to gauge healthy aging. However, because these data only use measurements at two age intervals, it remains unclear whether these biomarkers change monotonically with age. To address the relationship between each of the serum biomarkers we measured the Pearson's correlation, as shown in Table 2. Six biomarkers (MIP-1β, IP-10, IL-7, IL-12p40, PDGFβ and eotaxin) displayed robust r^2 ^values ≥ 0.75. To evaluate potential networks between these biomarkers, we utilized Ingenuity Pathway analysis (IPA) and observed that Akt, NFkB and TGFβ signaling were common pathways among five of these biomarkers, MIP-1β, IP-10, IL-7, IL-12p40, PDGFβ (data not shown), possibly suggesting that growth, inflammatory and fibrotic regulatory mechanisms, respectively contribute to the biomarker profile.

**Table 2:** Correlation between Baseline Markers.

	Leptin	IL5	MCP1	VEGF	PIIINP	PDGFBB	IGF1	Eotaxin	IL7	IP10	MIP1β	IL12(p40)
Leptin		0.50	-0.28	0.09	0.16	0.10	-0.26	0.02	0.11	-0.06	-0.11	-0.15
IL5	0.50		0.42	0.50	0.04	0.01	-0.23	0.00	0.09	0.02	-0.15	-0.18
MCP1	-0.28	0.42		-0.04	0.10	-0.07	-0.57	-0.30	-0.04	0.65	0.54	-0.06
VEGF	0.09	0.50	-0.04		-0.34	0.51	0.29	0.50	0.35	0.34	0.14	-0.43
PIIINP	0.16	0.04	0.10	-0.34		0.46	0.34	0.44	0.57	0.71	0.43	0.62
PDGFBB	0.10	0.01	-0.07	0.51	0.46		0.62	0.68	***0.85***	***0.79***	***0.82***	***0.85***
IGF1	-0.26	-0.23	-0.57	0.29	0.34	0.62		0.43	0.54	0.52	0.62	0.69
Eotaxin	0.02	0.00	-0.30	0.50	0.44	0.68	0.43		***0.75***	***0.82***	0.64	0.69
IL7	0.11	0.09	-0.04	0.35	0.57	***0.85***	0.54	***0.75***		0.69	0.67	0.65
IP10	-0.06	0.02	0.65	0.34	0.71	***0.79***	0.52	***0.82***	0.69		0.73	***0.80***
MIP1β	-0.11	-0.15	0.54	0.14	0.43	***0.82***	0.62	0.64	0.67	0.73		***0.89***
IL-12(p40)	-0.15	-0.18	-0.06	-0.43	0.62	***0.85***	0.69	0.69	0.65	***0.80***	***0.89***	

A pairwise Pearson's correlation was done to evaluate the relationship between biomarker levels in the young and old subjects combined. The r^2 ^values for each pairwise correlation is shown. The r^2 ^values ≥ 0.75 were used to identify networks using Ingenuity Pathway Analysis (IPA). In data not shown Akt, NFκB and TGFβ pathways were common among these biomarkers.

### Testosterone related response in serum biomarkers

**Figure 2:**
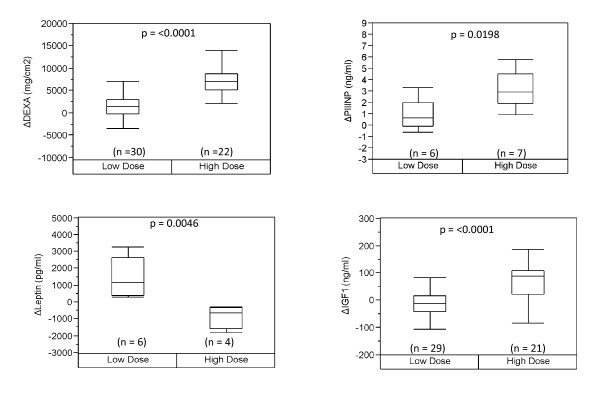
**Biomarker response based on testosterone dose in the young**. We evaluated the change in biomarkers that differ significantly between low and higher doses of testosterone in younger subjects. P-values are indicated based on a Student *t*-test.

**Figure 3:**
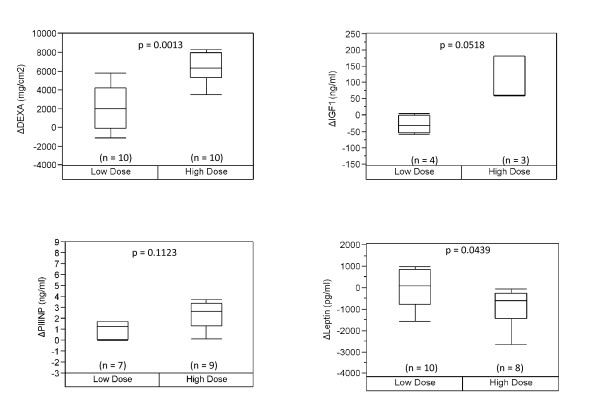
**Biomarker response based on testosterone dose in the older group**. We evaluated the change in biomarkers that differ significantly between low and higher doses of testosterone in older subjects. Leptin was found to be significant to a p-value of < 0.05, however both PIIINP and IGF1 showed a trend change. P-values are indicated based on a Student *t*-test.

**Figure 4:**
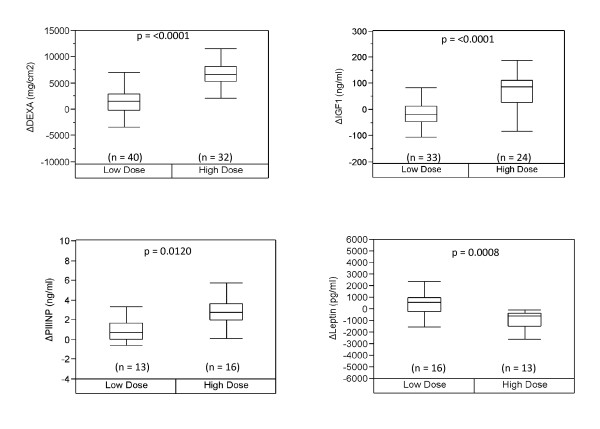
**Biomarker response based on testosterone dose in the young and old combined**. We evaluated the change in biomarkers that differ significantly between low and higher dose of testosterone in combined young and older subjects. Three factors in addition to DEXA were found to be significant to p-value less than 0.05. P-values are indicated based on a Student *t*-test.

Testosterone supplementation has been shown to increase muscle mass in both young and older men. We were interested in seeing if the factors affected by age were also responsive to testosterone supplementation and in finding markers for testosterone supplementation that are robust in both the young and the old. We evaluated the response to graded doses of testosterone in relation to a young age group (Figure 2), an older age group (Figure 3) as well as both groups combined (Figure 4). Due to the small sample size, we based our analysis on two groups, low dosage (25 mg per week to 125 mg per week) and high dosage (greater than 300 mg per week) of testosterone, looking at the change in biomarker levels between the baseline and the end of treatment. Based on this approach, we observed three serum factors measured in single-plex assays showed a significant change in association with testosterone dose in the young, with significance values of p < 0.05 for each independent assay. These include PIIINP, leptin, and IGF-1 (Figure 2). We also observed a significant difference with testosterone dosage in levels of ENA78 and MIG, however due to a small sample size (n = 2), these data are not shown. In the older men, only leptin and DEXA displayed a similar trend change as seen in the young men at a p-value of ≤0.05 for each independent assay (Figure 3). Notably, IGF1 and PIIINP showed a trend change but did not reach significance. This may be due to the small sample size of the older population and/or the sampling time of the baseline values (within first two weeks) in the young, since these biomarkers may have already begun to rise and thereby affect the change observed. When the data from older men and younger men were combined, we observed that three of the biomarkers seen in the young, i.e., IGF1, PIIINP and leptin, as well as DEXA, remained significant using a p-value of ≤0.05 for each independent assay (Figure 4). These data indicate that a subset of biomarkers associated with healthy aging men also change with testosterone administration, given the limits of detection for the assays used in this study. Whether a similar profile of anabolic change occurs with exercise or physical function improvements remains an important question in efforts to more fully understand the limits of androgen improvements and specificity. These data also represent the first comparative evaluation of biomarkers in young and old subjects receiving equivalent testosterone supplementation.

## Discussion

In this pilot study we describe serum proteins that change in association with healthy aging among men between the ages of 18 and 35 compared with older men between the ages of 60 and 85. We provide evidence that a subset of these biomarkers, but not all, appear to be responsive to anabolic administration. These data support the high likelihood of a serum profile for aging that may be useful for establishing response profiles for therapeutic androgen administration in older and younger men, particularly when measured against a reliable age reference group for baseline biomarker levels. Thus, the use of biomarkers in evaluating anabolic response would benefit from studies that precisely define the profile of biomarker change during healthy aging to adequately gauge the effect(s) of gains associated with anabolic therapy.

Of the serum biomarkers screened at baseline in both healthy young and older men, we provide evidence for nine that displayed differences in baseline levels at two different age groups: IGF1, PDGFBB, PIIINP, eotaxin, IP-10, IL-12(p40), IL-7, MIP1β and MIG. IGF1 levels, have been shown previously to decrease with increased age and have been associated with muscle declines [31]. PIIINP, a marker of remodeling and collagen synthesis has not been previously studied with aging. The declines in PIIINP levels in older men suggest a possible decrease in remodeling with age. The remaining immunoregulatory biomarkers collectively suggest a possible decrease in T-cell and neutrophil response with healthy aging that could influence muscle maintenance with aging [32-38]. Pathway analysis using IPA for signaling networks common to these biomarkers highlight a potential role for Akt, NFκ and TGFβ pathways, all of which have been independently associated with aging [39-42]. The correlated changes in these biomarkers suggest that perhaps a change in one of these markers may be predictive of changes in other networked biomarkers as an aggregate. However, this possibility of aggregate biomarker profiles will require further study with larger sampling than was available in this pilot study.

Previous studies on the effects of testosterone supplementation have indicated that similar gains in muscle strength and fat free mass are observed in older men compared with younger men [11,13]. Previous separate studies have addressed individual serum biomarkers that change in association with anabolic administration (e.g., leptin, IGF-1) in the young [11,22] and old [23,27]. Importantly, our study confirms a recent study showing that PIIINP levels increase with testosterone and predict gains in lean muscle mass in both young men and older men [15]. Although we do confirm concordant gains with some biomarkers, we also notice that despite similar gains in muscle strength and mass, older men differed from younger men in baseline levels of biomarkers and in the response profile of these markers associated with testosterone supplementation. This suggests, at least for this limited set of biomarkers, and in these subjects, that gains in muscle mass and potentially associated gains in physical function do not display simple concordance with the serum profile. This may suggest the existence of complex mechanisms for testosterone response that will require further study. Additional biomarkers that accurately reflect anabolic response would assist in more accurately defining anabolic therapeutic outcomes as well as other approaches for gains in muscle mass and function, *e.g.*, exercise and other function promoting therapeutics. The direct and combined comparative study described in this report validates the robustness and non-equivalency of multiple markers in association with dose and age - an observation that has not been fully recognized to date.

Finally, previous studies have shown that testosterone levels generally decline with age in men [17,43]. Whether there is a feedback relationship between age dependent declines in testosterone and biomarkers is beyond the scope of this study but remains an intriguing possibility. The dampened biomarker profile observed with an older age group compared to a younger age group in this study suggests that testosterone's effect on anabolic pathways may change with age while nevertheless achieving the same gains in mass and strength. Future studies evaluating age- and anabolic-dependent shifts in biomarker levels will assist in identifying novel pathways that can be used to promote gains in muscle mass across a broad age range.

## Competing interests

The authors declare that they have no competing interests.

## Authors' contributions

CB drafted the manuscript, ran assays and analyzed the data. JU ran the RIA and helped analyze data for PIIINP experiments. ED, MSM and RJU participated in the design and coordination of the questions asked using the Texas cohort. QD and BF ran assays and drafted figures and tables. TS and SB conceived concept and provided samples and data for the Boston cohort. PS provided statistical analysis. MM conceived the questions asked overall in this study and participated in the design, coordination and writing of the manuscript. All authors have read and approved this manuscript.
